# Intubating conditions and side effects of propofol, remifentanil and sevoflurane compared with propofol, remifentanil and rocuronium: a randomised, prospective, clinical trial

**DOI:** 10.1186/1471-2253-14-39

**Published:** 2014-05-22

**Authors:** Thomas Mencke, Refa Maria Jacobs, Susann Machmueller, Martin Sauer, Christine Heidecke, Anja Kallert, Hans Wilhelm Pau, Gabriele Noeldge-Schomburg, Attila Ovari

**Affiliations:** 1 Department of Anaesthesia and Intensive Care Medicine, University of Rostock, Schillingallee 35, Rostock 18057, Germany; 2 Department of Otorhinolaryngology, University of Rostock, Rostock, Germany

**Keywords:** Sevoflurane, Propofol, Remifentanil, Neuromuscular block, Intubating conditions, Laryngeal injury

## Abstract

**Background:**

Tracheal intubation without muscle relaxants is usually performed with remifentanil and propofol or sevoflurane. Remifentanil 1.0 to 4.0 μg·kg^-1^ and propofol 2.0-3.0 mg·kg^-1^ or sevoflurane up to 8.0 Vol% provide acceptable, i.e. excellent or good intubating conditions. We hypothesized that sevoflurane 1.0 MAC would provide acceptable intubating conditions when combined with propofol and remifentanil.

**Methods:**

Eighty-three patients to be intubated were randomised to two groups. The SEVO group received propofol 1.5 mg kg^-1^, remifentanil 0.30 μg kg min^-1^ and sevoflurane 1.0 MAC; the MR group received the same doses of propofol and remifentanil plus rocuronium 0.45 mg kg^-1^. We evaluated intubation and extubation conditions, mean arterial pressure (MAP), heart rate (HR) and bispectral index (BIS). The vocal cords were examined for injury by videolaryngoscopy before and 24 hours after surgery.

**Results:**

Acceptable intubating conditions were seen more frequently with rocuronium than with sevoflurane: 97% versus 82%; p = 0.03; the subscore for vocal cords was comparable: 100% versus 98%. MAP before intubation decreased significantly compared with the MAP at baseline to the same extent in both groups; ephedrine IV was given in 15 (SEVO) versus 16 (MR) patients; p = 0.93. BIS at tracheal intubation was 27 (13-65) in the SEVO group, 29 (14-62) in the MR group; p = 0.07. Vocal cord injuries (oedema, haematoma) were similar: 4 patients in each group.

**Conclusions:**

Overall intubating conditions were better when rocuronium was used; the subscore for vocal cords was comparable. The incidence of side effects was the same in the two groups.

**Trial registration:**

ClinicalTrials.Gov: NCT 01591031.

## Background

Tracheal intubation without neuromuscular blocking agents (NMBAs) is performed mainly with remifentanil and propofol [1,2]; alfentanil, fentanyl and sufentanil are alternative opioids [3-5]. Alternative hypnotic agents are etomidate, thiopental and sevoflurane [6,7]. Because poor intubating conditions are associated with vocal cord injury [3] it is important to achieve good or excellent, i.e., acceptable, intubating conditions. Remifentanil, 1.0 to 4.0 μg·kg^-1^ combined with propofol, provides acceptable intubating conditions [1,2,4], but these doses may cause arterial hypotension and severe bradycardia [7,8]. The remifentanil infusion rate can be reduced when a volatile anaesthetic such as isoflurane is added rather than propofol [9]. Sevoflurane 8 Vol% (i.e., 4.0 minimal alveolar concentration; MAC) combined with remifentanil 2.0 μg·kg^-1^ provided acceptable intubating conditions in 97% of patients, but mean arterial pressure (MAP) decreased by about 25% [7]. We hypothesized that sevoflurane 1.0 MAC would provide clinically acceptable intubating conditions, when combined with a standard anaesthesia induction with propofol and a continuous infusion of remifentanil.

## Methods

The SEVO (sevoflurane) group received propofol, remifentanil and sevoflurane 1.0 MAC; the MR (muscle relaxant) group received propofol, remifentanil and rocuronium 0.45 mg·kg^-1^. The primary outcome measure was the intubation score [10]. Secondary outcome measures were MAP, heart rate (HR) and bispectral index (BIS); in addition, we assessed the incidence and severity of vocal cord injuries at 24 hours after surgery by videolaryngoscopy, the incidence and severity of hoarseness and sore throat up to 72 hours after surgery.

### Ethics approval and registration

This prospective, randomised, clinical study was conducted between April 2012 and January 2013 at the University Hospital of Rostock, Germany. Ethical approval was provided by the Institutional Review Committee (Ethikkommission der Universitaet Rostock, Rostock, Germany; registration number: A 2011 124) on 3 November 2011. The study was registered at ClinicalTrials.Gov with the number NCT 01591031.

### Inclusion and exclusion criteria

After obtaining written informed consent, we studied 88 patients, American Society of Anesthesiologist (ASA) grade I-III aged 18-80 years, who required orotracheal intubation for ear surgery. Exclusion criteria were a known or suspected difficult airway, such as mouth opening < 3.5 cm or Mallampati score 4 or Cormack grade 3 and 4; obesity; disease of the larynx or vocal cords; hoarseness before surgery; and a preexisting severe vocal cord pathology discovered at videolaryngoscopy by an ear-nose-throat (ENT) physician before surgery.

### Randomisation and monitoring

A randomisation program was used [11]. Patients in group MR were monitored with acceleromyography to achieve maximum neuromuscular block at the time of tracheal intubation in the MR group, i.e, when the TOF counts disappeared. Neuromuscular monitoring was performed with the TOF Watch SX® device (Organon Teknika, Eppelheim, Germany). Neuromonitoring was performed in all with the BIS Vista® brain monitoring system (Aspect Medical Systems, Norwood, MA, USA). It was used to measure the depth of anaesthesia at time of tracheal intubation. Blood pressure was measured non-invasively every 2 minutes during induction of anaesthesia and afterwards every 5 minutes.

### Induction and maintenance of anaesthesia

All patients received midazolam 7.5 mg orally before arrival in the anaesthetic room, where they were given dexamethasone 4.0 mg IV and ondansetrone 4.0 mg IV. Induction of anaesthesia was standardised: all patients received remifentanil 0.30 μg·kg·min^-1^ for three minutes, after which propofol 1.5 mg·kg^-1^ was given (if necessary 30 mg was supplemented). After the propofol, the SEVO group received sevoflurane at an inspired concentration of 3.0-3.5 Vol% (fresh gas flow 8 l·min^-1^). After 2-3 minutes, when the endtidal sevofluane concentration reached 1.0 MAC (stable for 20 s), intubation was performed. MAC was calculated from the age of the patient with the software from the Primus® anaesthetic machine (Dräger Medical Germany, Lübeck, Germany). The sevoflurane was discontinued after intubation. In the MR group, after propofol, rocuronium 0.45 mg·kg^-1^ was given after calibration of the TOF watch SX. If maximum block did not occur, a further 0.15 mg·kg^-1^ rocuronium was added. The trachea was intubated when maximum block was achieved, by the same anaesthesiologist each time.

Maintenance of anaesthesia was by propofol 4.0 – 6.0 mg·kg·h^-1^ and remifentanil 0.20-0.30 μg·kg·min^-1^ in both groups. Ephedrine 5-10 mg IV was given if systolic pressure decreased by 20% and/or below 100 mmHg and atropine IV if the HR decreased below 45 min^-1^. The patients were extubated when they opened their eyes and/or began to cough. They were then moved to the postanaesthesia care unit (PACU).

### Intubating conditions, intubating variables and extubating conditions

Intubating conditions were assessed according to the GCRP guidelines (see Table 1) [10]. Extubating conditions assessed during removal of the tracheal tube were defined as follows: excellent = no coughing and no movement of the limbs, good = slight coughing or slight movement of the limbs or both; poor = sustained coughing and/or vigorous movement of the limbs during extubation. Intubating variables were assessed: application of back upward right pressure (BURP), Cormack and Lehane grade, time for intubation and number of intubation attempts (see Appendix). We used RAE tubes (Mallinckrodt®, Covidien, Dublin, Irland) standardized to 8.0 mm for men and 7.0 mm for women. Cuff pressure was measured and adjusted continuously below 25 cm H_2_0 by a cuff pressure monitor. No gastric tubes or intubation stylets were used.

**Table 1:** Scoring conditions for tracheal intubation

	**Intubating scores**		
	**Clinically acceptable**		**Clinically not acceptable**
**Variable**	**Excellent**	**Good**	**Poor**
**Laryngoscopy**			
Jaw relaxation	Relaxed	Not fully	Poor
Resistance to laryngoscope	None	Slight	Active
**Vocal cords**			
Position	Abducted	Intermediate	Closed
Movement	None	Moving	Closing
**Reaction to tube insertion or cuff inflation**			
Movement of limbs	None	Slight	Vigorous
Coughing	None	Slight	Sustained

Intubating conditions: excellent = all qualities are excellent; good = all qualities are excellent or good; poor = any quality is poor. Excellent and good intubating conditions are summarised as clinically acceptable conditions [10].

### Hoarseness and sore throat

Hoarseness and sore throat were assessed in the PACU and daily up to 72 hours by a physician blinded to the patient’s group assignment. The incidence and severity of hoarseness (defined as change in the acoustic quality) and sore throat (defined as a continuous throat pain) were assessed according to 4 point scales (see Appendix) [12,13].

### Vocal cord injuries

All patients underwent laryngoscopy before surgery by an ENT physician who was blinded to the patient’s group. Slight changes, such as erythema, and vocal cord injuries, such as oedema, haematoma and granuloma by videolaryngoscopy (see Appendix) were noted. The recordings before and after surgery were compared; any changes were assessed as to the possibility that they could be caused by intubation.

### Statistical analysis

We used the SigmaStat® for Windows Version 3.5, (Systat Sotware Inc., San Jose, California, USA). Demographic data were analysed using Mann-Whitney *U*-test or *t*-test. Comparisons between the study groups were performed using the χ^2^test, Fisher’s exact test or Kruskal-Wallis ANOVA test. Results were considered statistically significant when p < 0.05.

A sample size calculation assumed an incidence of acceptable intubating conditions of 100% in the rocuronium group and 75% in the sevoflurane group. Therefore we needed 66 patients (33 patients in each group; α = 0.05, 1-β = 80%).

## Results

**Figure 1:**
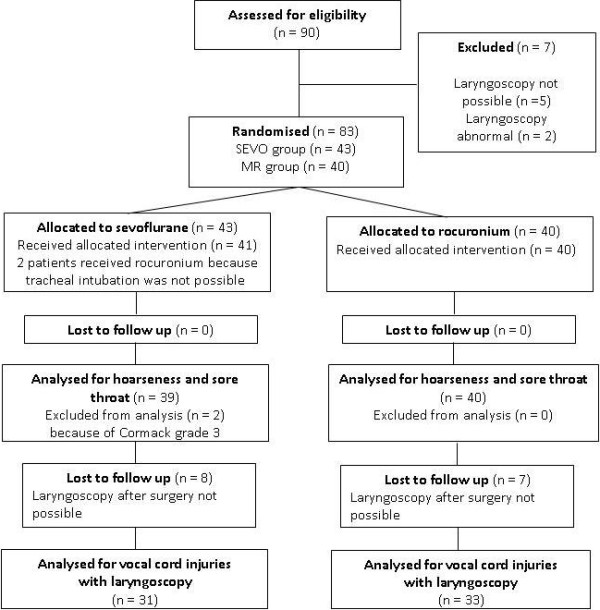
Flow chart of patient distribution.

We randomised 83 patients; 43 to the SEVO group and 40 to the MR group (see Figure 1). Patient characteristics were comparable (Table 2). BMI was less, but not significantly in the SEVO group (p = 0.06). The duration of anaesthesia was significantly greater in the MR group (p = 0.01). BIS values and use of ephedrine were similar (Table 2). Table 3 shows the haemodynamic response to tracheal intubation.

**Table 2:** Patient characteristics, duration of anaesthesia, BIS and ephedrine administration

	**SEVO group**	**MR group**	**P**
	**(n = 39)**	**(n = 40)**	
Age (yr)	48 ± 17	50 ± 16	0.67
Weight (kg)	79.6 ± 15	83.8 ± 16	0.23
Height (cm)	173 ± 11	172 ± 9	0.70
Body mass index (kg·m^-2^)	26.5 ± 3.7	28.2 ± 4.3	0.06
Gender ratio (female/male)	16/23	16/24	0.89
ASA I/II/III	5/30/4	3/33/4	0.73
Duration of anaesthesia (min)	81 (38-193)	121 (48-214)	0.01
BIS at tracheal intubation	27 (13-65)	29 (14-62)	0.07
BIS at tracheal extubation	72 (43-91)	71 (42-88)	0.89
Administration of ephedrine (n)	15 (38)	16 (40)	0.93

Values are mean ± SD, or numbers (%) or median (range). BIS = bispectral index.

SEVO group = Induction of anaesthesia with sevoflurane.

MR group = Induction of anaesthesia with rocuronium.

**Table 3:** Induction of anaesthesia and haemodynamic response to tracheal intubation

	**SEVO group**	**MR group**	**P**
	**(n = 39)**	**(n = 40)**	
Mean arterial pressure (mmHg)			
Preinduction	92 ± 11	95 ± 11	0.18
Postinduction	65 ± 9*	65 ± 8*	0.98
Postintubation	67 ± 6*	68 ± 9*	0.34
Heart rate (beats/min)			
Preinduction	71 ± 12	77 ± 14	0.05
Postinduction	63 ± 10*	65 ± 11*	0.51
Postintubation	60 ± 10*	65 ± 10*	0.03

Values are mean ± SD. *p < 0.05 versus preinduction values.

Preinduction = before induction of anaesthesia; Postinduction = 3 min after induction of anaesthesia but before tracheal intubation; Postintubation = 2 min after tracheal intubation.

SEVO group = Induction of anaesthesia with sevoflurane.

MR group = Induction of anaesthesia with rocuronium.

### Intubating conditions, intubating variables and extubating conditions

**Figure 2:**
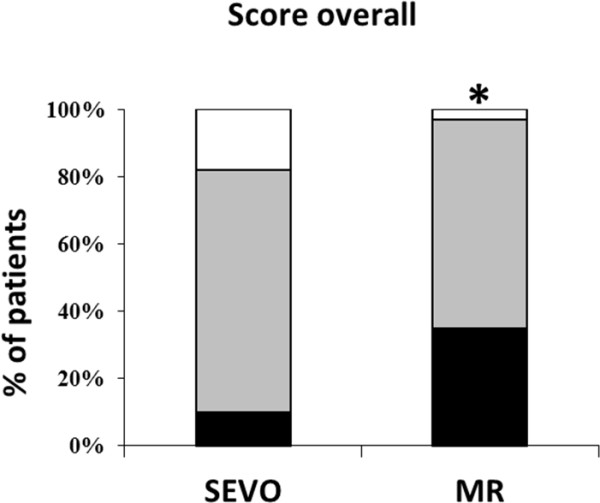
**Patients (%) with excellent (black bars), good (grey bars) and poor intubation conditions (white bars).** *p = 0.06 MR group versus SEVO group overall; p = 0.029 clinically acceptable (excellent and good) conditions. SEVO group = Induction of anaesthesia with sevoflurane. MR group = Induction of anaesthesia with rocuronium.

**Figure 3:**
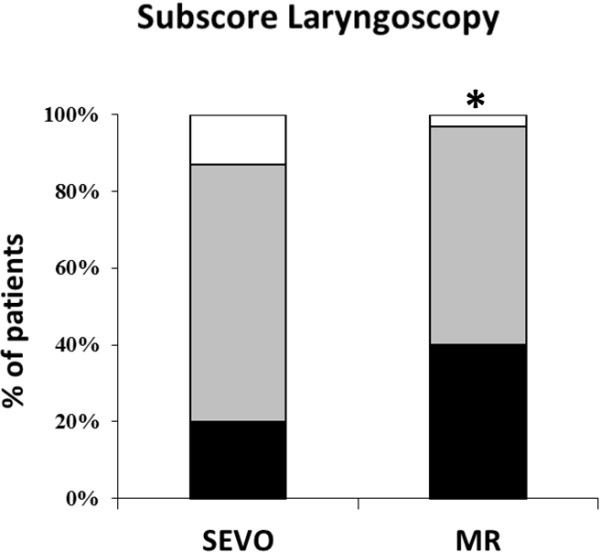
**Patients (%) with excellent (black bars), good (grey bars) and poor intubation conditions (white bars) for subscore laryngoscopy.** *p = 0.06 MR group versus SEVO group. SEVO group = Induction of anaesthesia with sevoflurane. MR group = Induction of anaesthesia with rocuronium.

**Figure 4:**
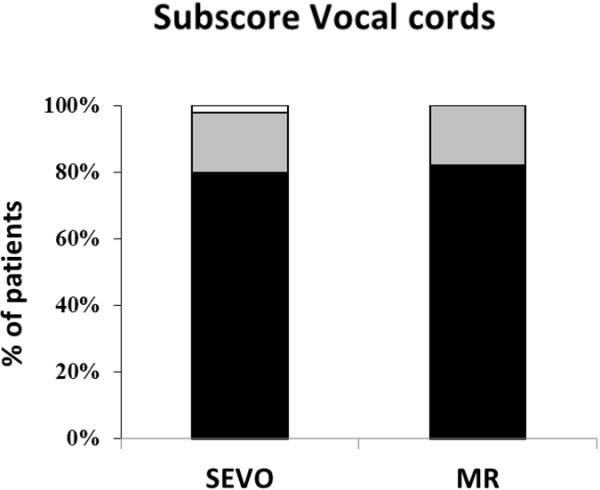
**Patients (%) with excellent (black bars), good (grey bars) and poor intubation conditions (white bars) for subscore vocal cords.** MR group versus SEVO group: p = 0.59. SEVO group = Induction of anaesthesia with sevoflurane. MR group = Induction of anaesthesia with rocuronium.

**Figure 5:**
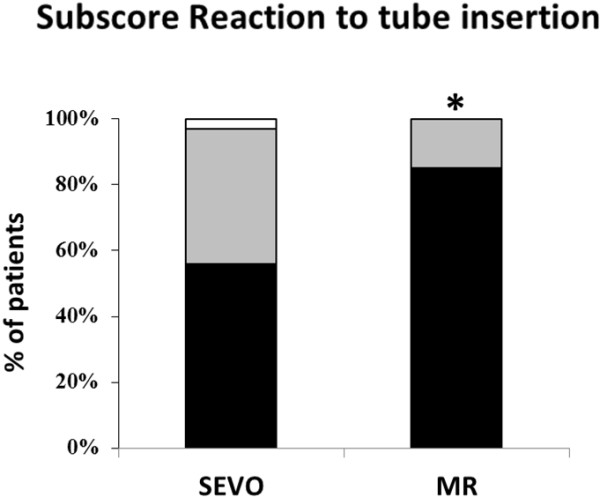
**Patients (%) with excellent (black bars), good (grey bars) and poor intubation conditions (white bars) for subscore reaction to tube insertion or cuff inflation.** *p = 0.02 MR group versus SEVO group. SEVO group = Induction of anaesthesia with sevoflurane. MR group = Induction of anaesthesia with rocuronium.

Tracheal intubation was possible in all patients, but two patients from the SEVO group could only be intubated after administration of rocuronium (see Figure 1). The vocal cords were closed and did not open after propofol 30 mg IV; to avoid vocal cord injuries, rocuronium 0.45 mg·kg^-1^ was given. Overall intubating conditions were not significantly better in group MR (p = 0.06). However, acceptable intubating conditions (excellent and good) were significantly more common (p = 0.03; Figure 2). The subscores for the vocal cords were not significantly different, but the subscore reaction to tube insertion or cuff inflation was significantly better in group MR (p = 0.02; Figures 3, 4 and 5). Intubating variables and extubating conditions are shown in Table 4.

**Table 4:** Intubating variables and conditions during tracheal extubation

	**SEVO group**	**MR group**	**P**
	**(n = 39)**	**(n = 40)**	
Intubating variables			
Mallampati I/II/III	20/19/0	18/21/1	0.55
Cormack grades 1/2	26/13	24/16	0.70
Application of BURP	8	12	0.48
Time for intubation (s)	21 ± 17	19 ± 12	0.44
Attempts (n) 1/2	36/3	35/5	0.71
Extubating conditions			
Coughing and movement of limbs excellent/good/poor	1/21/17	3/26/11	0.25

Values are mean ± SD or numbers. BURP = back upward right pressure.

SEVO group = Induction of anaesthesia with sevoflurane.

MR group = Induction of anaesthesia with rocuronium.

### Hoarseness and sore throat

The overall incidence of hoarseness was 21.5%, with no significant difference between the groups. The overall incidence of sore throat was 52%, again with no significant difference between groups. No patient suffered from hoarseness or sore throat exceeding 72 hours.

### Vocal cord injuries

The overall incidence of vocal cord injuries was 12.5% (Table 5). There were two haematomas and six cases of oedema, but no granulomas. Slight changes, such as erythema, were found in 13 patients in the SEVO group and eight in the MR group (n.s.).

**Table 5:** Vocal cord injuries and erythema

	**SEVO group**	**MR group**	**P**
	**(n = 31)**	**(n = 33)**	
Incidence of injuries			
Haematoma, oedema	4 (13)	4 (12)	1.00
Granuloma	0	0	-
Slight changes			
Erythema	13 (42)	8 (24)	0.21
Side (erythema and injuries)			
Bilateral	16	11	0.22
Left	0	1 (erythema)	
Right	1 (hematoma)	0	

Values are numbers (%).

SEVO group = Induction of anaesthesia with sevoflurane.

MR group = Induction of anaesthesia with rocuronium.

## Discussion

Tracheal intubation with propofol 1.5 mg·kg^-1^, remifentanil 0.30 μg·kg^-1^·min^-1^ and sevoflurane 1.0 MAC was feasible and safe, but intubating conditions were not as good as in the rocuronium group. Acceptable intubating conditions were present in 82% of the patients in the SEVO group, and the subscore for the vocal cords was acceptable in 98%. MAP and ephedrine application were similar in the two groups, as were vocal cord injuries.

Tracheal intubation without NMBAs is performed more frequently, especially in ambulatory surgery [14]. In Germany in 2005, 20% of elective tracheal intubations were performed without NMBAs [15]. The laryngeal mask offers an alternative, but for surgery in the prone position, laparoscopic or ENT surgery, tracheal intubation is essential [16]. Intubation without NMBAs avoids postoperative residual blockade and allergic reactions to muscle relaxants.

Combining propofol with remifentanil, 1.0 to 4.0 μg·kg^-1^ provided acceptable intubating conditions [1,2,4,17]. Remifentanil 2 μg·kg^-1^ and propofol 2.0 mg·kg^-1^ were sufficient to obtain excellent intubating conditions in 11 of 12 healthy volunteers [17]. The drugs were given over 5 to 10 seconds, the propofol immediately after the remifentanil, a method which is safe in healthy volunteers or young patients, but not for patients of ASA grade II or III. As much as 4.0 μg·kg^1^ may be necessary to obtain excellent intubating conditions [18]. Remifentanil ≥ 2 μg·kg^-1^ is not suitable for old patients or those with cardiovascular disease because it is associated with arterial hypotension and bradycardia [18]. In one observational study the choice of agent depended on the decision of the anesthesiologist [5]. Acceptable intubating conditions were found in 98.2% of the patients in the relaxant-free intubating group; post-intubation laryngeal symptoms like sore throat and dysphonia were comparable between groups. In the relaxant-free group the median dosage of propofol was 3.64 mg·kg^-1^, supplemented with sufentanil [5]. Arterial hypotension was observed in 14% of patients, to a similar degree in the two groups. Patients in the relaxant-free group, however, were significantly younger and in better ASA grades [5]. Others found that when NMBAs are omitted, difficult intubation is more common [19].

Significantly fewer patients in our SEVO group had acceptable intubating conditions. The subscore vocal cords, however, was acceptable in all SEVO patients except one. Sevoflurane relaxes the bronchial muscles and possibly also the laryngeal muscles [20]. The other subscores, however, were significantly worse in the SEVO group.

Sevoflurane has been used as the sole agent for tracheal intubation; the ED_95_ for tracheal intubation was 8.07% (end-tidal concentration) [21]. Conditions after induction with sevoflurane 6% and N_2_O 66% in O_2_ were comparable to those with succinylcholine 1.5 mg·kg^-1^[22]. This technique has been proposed for patients in whom succinylcholine is contraindicated [22]. Mean arterial pressure and heart rate were significantly lower immediately before tracheal intubation with sevoflurane. Adding remifentanil 2.0 μg·kg^-1^ to sevoflurane 8 Vol% resulted in acceptable intubating conditions in 29 of 30 patients [7], a similar incidence to intubating conditions with rocuronium 0.6 mg·kg^-1^[23]. Cros et al. applied a modified Dixon’s up-and-down method to remifentanil 1.0 μg·kg^-1^ followed by an infusion of 0.25 μg·kg^-1^·min^-1^, increasing or decreasing sevoflurane in 0.5% steps. The concentration of sevoflurane for acceptable intubating conditions was 2.5 ± 0.7% [24]. Sevoflurane 8 Vol% combined with remifentanil 2.0 μg·kg^-1^ caused a significant reduction of mean arterial pressure in young ambulatory patients (median age 16 and 18 years) [7]. In our study, MAP decreased in both groups by about 30%, and ephedrine use was similar. MAP decreased more than expected in our patients (mean ages 48 and 50 years), who were much older than those of Cagiran et al. [7].

Our study included patients of ASA III grade with coronary artery and cerebrovascular disease. The heart rate was significantly lower after tracheal intubation with sevoflurane, which is advantageous for patients with coronary heart disease.

Avoiding neuromuscular blockade may increase the risk of difficult mask ventilation [25,26]. In a Danish observational study, tracheal intubation was difficult in 5.1% of 103,812 patients; avoiding neuromuscular blockade was one risk factor in the multivariate analysis (odds ratio 1.48) [25]. Neuromuscular blockade with rocuronium facilitated mask ventilation significantly compared with saline; in all 42 patients with rocuronium, ventilation was possible by mask [26]. In an observational study of 53041 patients in whom mask ventilation was attempted, it was impossible in 77 (0.15%); 73 patients had received a neuromuscular blocking drug during management of the airway and neuromuscular blockade did not improve matters [27]. Changes caused by radiotherapy of the neck were the most important risk factor for impossible mask ventilation [27]. Difficult mask ventilation combined with difficult laryngoscopy was observed in 698 patients (0.40%) from 176,679 patients [28]. Independent risk factors for difficult mask ventilation combined with difficult laryngoscopy included radiotherapy changes, the presence of teeth, Mallampati III or IV and male sex. The impact of neuromuscular blocking agents could not be assessed [28].

Tracheal intubation with propofol and fentanyl alone was associated with a greater frequency and severity of hoarseness and vocal cord injuries compared with a group receiving these drugs and atracurium; intubating conditions were better with atracurium [3]. Without rocuronium, there was more hoarseness and intubating conditions were worse [17]. Nevertheless, Bouvet et al. showed that propofol and remifentanil were associated with a similar incidence of hoarseness and vocal cord injuries to cisatracurium [29], although fibroscopic examination was done 48 hours after surgery only in patients with persisted hoarseness; moreover, small tubes with an ID of 6.5 or 7.0 mm were used [29]. The incidence of vocal cord injuries in patients receiving NMBAs was up to 27%, and 42% in patients receiving propofol and fentanyl without NMBAs [3,30-33]. In the present study, however, we used remifentanil instead of fentanyl, and also sevoflurane. Vocal cord injuries such as haematoma and oedema were present in our study in 12.5%; the incidence of erythema was comparable between groups. We suppose that sevoflurane contributed to this low incidence of vocal cord injuries because the intubating conditions at the vocal cords were clinically acceptable in 98%. Obregon et al. showed that adding sevoflurane to propofol and remifentanil gave the same incidence of hoarseness as propofol, remifentanil and rocuronium [34]. We confirmed these results; in addition, we showed that laryngeal injuries were not more common under sevoflurane anaesthesia. Vocal cord injuries may occur during tracheal intubation, during surgery and at the end of anaesthesia, when the tube is removed. Therefore, we assessed not only intubating conditions, but also extubating conditions to reveal possible risk factors for vocal cord injuries. The duration of anaesthesia was significantly greater in the MR group, which was unexpected as the patients were randomised. Duration of anaesthesia exceeding five hours is a risk factor for hoarseness or sore throat [35,36]; our anaesthetics lasted less than four hours. The head was not moved during ear surgery; therefore, we suppose that duration of anaesthesia had no additional effect on laryngeal morbidity.

Our study has two more limitations. Firstly, it did not include any patients with the criteria of difficult intubation because we wanted to have comparable patients in both groups. Second, the investigator who assessed conditions at intubation could not be blinded as to the presence of sevoflurane. However, the investigator who studied hoarseness and sore throat up to 72 hours was blinded as was the ENT physician who performed the videolaryngoscopy.

## Conclusions

In conclusion, we showed that intubating conditions with propofol 1.5 mg·kg^-1^, remifentanil 0.30 μg·kg^-1^·min^-1^ and sevoflurane 1.0 MAC-instead of rocuronium 0.45 mg·kg^-1^-were acceptable in 82% of patients although conditions were better with rocuronium. The absence of a neuromuscular blocking agent did not affect the incidence and severity of vocal cord injuries.

## Appendix

### Hoarseness, sore throat and vocal cord pathologies

A. Hoarseness [3]

Do you have any hoarseness?

No = 0; yes was graded as follows:

1 = noticed by patient, 2 = obvious to observer, 3 = aphonia.

B. Sore throat [13]

Do you have any sore throat?

No = 0; if yes, sore throat was graded as follows: 1 = mild (pain with deglutition), 2 = moderate (pain present constantly and increasing with deglutition), 3 = severe (pain interfering with eating and requiring analgesic medication).

C. Vocal cord pathologies [3,30,33]

Location: unilateral (left or right vocal cord) or bilateral (both vocal cords).

Morphology:

– Slight changes: erythema = redness of the mucosa.

– Vocal cord injuries: oedema = swollen mucosa at the vocal folds, haematoma = bleeding into vocal cord, granuloma = granulation tissue remains as chronic, localised, rounded tissue.

D. Intubating variables

– Time for intubation = time in seconds from the initial inserting of the laryngoscope into the patient’s mouth until inflation of the cuff.

– Number of intubating attempts.

## Competing interests

There was no financial support or sponsorship. None of the authors has any conflict of interest.

## Authors’ contribution

TM made substantial contributions to conception and design, acquisition of data, and statistical analysis and interpretation of data; he drafted the article and he approved the final version to be published. RMJ made substantial contributions to conception and design; she performed the tracheal intubations; she revised the article critically for important intellectual content; and she approved the final version of the manuscript. SM made substantial contributions to the conception and to the design and acquisition of data; she revised the manuscript before submission and approved the final version. CH made substantial contributions to design and acquisition of data; she performed the videolaryngoscopy, revised the article critically for important intellectual content, and approved the final version of the manuscript. AK made substantial contributions to acquisition of data and interpretation of data; she performed the videolaryngoscopy and revised the article before submitting. MS made substantial contributions to conception and design, acquisition of data, and interpretation of data, revised the article critically for important intellectual content, and approved the final version. HWP made substantial contributions to conception and design and analysis of data; he revised the article and approved the final version. GNS made substantial contributions to conception and design and interpretation of data, revised the manuscript and approved the final version. AV made substantial contributions to conception and design, collected and analysed data, performed the videolaryngoscopy, revised the manuscript for important intellectual content, and approved the final version of the manuscript before submitting it. All authors read and approved the final manuscript.

## Pre-publication history

The pre-publication history for this paper can be accessed here:

http://www.biomedcentral.com/1471-2253/14/39/prepub
